# Response differences in seed germination and seedling growth of three Asteraceae plants under saline-alkali stress

**DOI:** 10.7717/peerj.21814

**Published:** 2026-10-23

**Authors:** Shanlin Yang, Xueying Yang, Renjie Li, Yan Ji, Yuting Hu, Dingxu Jin, Renyue Wei, Yuwei Liu

**Affiliations:** College of Landscape Architecture, Changchun University, Changchun, Jilin, China

**Keywords:** Asteraceae, Germination indices, Seedling growth indices, Salt stress, Alkali stress

## Abstract

Soil salinization severely restricts plant growth and threatens ecological sustainability, particularly in soda saline-alkali regions such as the Songnen Plain. Asteraceae plants are widely used in vegetation restoration due to their strong ecological adaptability. In this study, *Tagetes erecta* L., *Cadendula officinalis* L., and *Leucanthemum maximum* (Ramood) DC were selected as experimental materials. Two types of stress (salt and alkali) were applied at five concentration levels each (excluding the distilled-water control), which respectively constituted a salinity gradient and an alkalinity gradient. Seed germination parameters, seedling growth traits, and physiological indicators were measured, and field validation was conducted in areas with mild, moderate, and severe saline-alkali soils in the Songnen Plain. *T. erecta* L. showed optimal germination at the low concentration of salt and alkalinity (25 mmol/L), exhibiting a “low promotion and high inhibition” pattern, and maintained relatively high vigor under high stress. *L. maximum* had the highest germination rate under non stress conditions but was highly sensitive to increasing salt and alkalinity, while its root growth remained relatively stable under salt stress. *C. officinalis* L. was highly sensitive to alkali stress. Alkali stress consistently exerted stronger inhibitory effects on seed germination and seedling growth than salt stress. Field validation further demonstrated that *T. erecta* L. maintained a significantly higher survival rate under severe saline-alkali conditions (HS) compared to *L. maximum* and *C. officinalis* L. (*p* < 0.05). This study identified interspecific differences in saline-alkali tolerance among three Asteraceae species, offering a data basis and scientific reference for plant selection and breeding in ecological restoration and landscaping of saline-alkali areas.

## Introduction

With its growing severity, soil salinization has now become a global issue affecting resources and ecosystems (Dos Santos et al., 2022; Ondrasek et al., 2022; Liu, Jiang & Yuan, 2024). In 2010, the global area of saline–alkali land exceeded 950 million hm^2^, and the total area of such land in China accounted for approximately 4% of the global total (Corwin & Yemoto, 2020). Saline-alkali stress leads to reduced crop yield and lower land use efficiency, which severely constrains agricultural sustainability and food security in China (Fang, Hou & Liang, 2021). The developmental stage of plants critically determines their sensitivity to salt stress: early ontogenetic phases, such as seed germination and seedling establishment, are particularly vulnerable to elevated salinity (Waheed et al., 2024). Therefore, investigating the effects of saline-alkali stress on seed germination and seedling growth is a crucial step in achieving sustainable utilization of salinized land, with vegetation restoration and ecological reconstruction at its core.

Seeds are the propagules of plants and form the foundation for plant growth and development (Li et al., 2025). Seed germination directly determines a species’ reproductive capacity under adversity, influencing the survival and developmental trajectory of plant populations (Xu et al., 2021). Such disturbances reduce seed germination vigor, delay emergence, damage the embryo, and inhibit vegetative growth, ultimately curtailing organic matter accumulation and crop yield (Atta et al., 2023). Investigating the saline-alkali tolerance mechanisms and response patterns of plant seeds during the germination stage is of critical scientific importance for understanding the early-stage strategies of plant adaptation to stress and assessing their colonization potential in saline-alkali lands.

Asteraceae is one of the largest families among angiosperms, with approximately 2,000 species distributed in China (Xie et al., 2023). Beyond their remarkable species diversity, numerous Asteraceae members hold substantial economic importance, including globally cultivated oil crops (*Helianthus annuus*), widely grown ornamental and medicinal plants (*Chrysanthemum morifolium*), leafy vegetables (*Lactuca sativa*), and medicinal herbs such as *Artemisia argyi* (Kong et al., 2023). Many Asteraceae species exhibit broad ecological adaptability and considerable stress resistance potential, making them a focus of attention for the restoration and utilization of saline-alkali lands (Liu & Zhai, 2025). As pioneer species in degraded environments, certain salt-tolerant Asteraceae plants achieve bioremediation through unique physiological mechanisms, thereby providing crucial support for the establishment of ecological barriers (Jing et al., 2024). While saline-alkali stress generally inhibits seed germination in Asteraceae plants, systematic comparisons of the response differences and stress tolerance thresholds among different species-particularly those with economic relevance-remain limited, which constrains their precise application in saline-alkali land restoration (Dong et al., 2021).

This study selected three herbaceous species from Asteraceae as experimental materials: *Tagetes erecta* L., *Calendula officinalis* L., and *Leucanthemum maximum* (Ramood) DC. All three species are widely cultivated across China, particularly in the northeastern region, as ornamental and medicinal plants, and have demonstrated a certain degree of ecological adaptability (Peng et al., 2022). *C. officinalis* L. exhibits significant tolerance to poor soils and low maintenance requirements; *T. erecta* L. is easy to cultivate for ground cover and can be used in biological pest control; *L. maximum* is capable of improving soil conditions and purifying pollutants (Liu et al., 2021; Jing et al., 2022). By subjecting them to salt stress and alkali stress, this research systematically compares the response differences in seed germination and seedling growth among these three Asteraceae plants. It aims to explore their saline-alkali tolerance capacities, thereby providing practical references for urban landscaping and ecological restoration in saline-alkali regions. Furthermore, it seeks to offer a scientific basis for selecting suitable plant materials for ecological rehabilitation in saline-alkali lands.

## Materials & Methods

### Study area

The Songnen Plain (43°36′–49°26′N, 121°21′–128°18′E) is the largest concentrated area of soda saline-alkali land in China. The saline-alkali land in Jilin Province is primarily distributed in the western part of the Songnen Plain, accounting for 62.46% of the national total. This includes approximately 2.89 million hm^2^ of strongly alkaline soil (pH > 9.5), posing a serious threat to agriculture (Qu, Chen & Wang, 2025; Shahrokhnia & Wu, 2021). This field experiment was performed in Tongyu, Qian’an, and Zhenlai on the Songnen Plain. Seeds of the three experimental materials were all collected from the Changbai Mountain National Nature Reserve in the southeastern part of Jilin Province, China (127°42′–128°16′E, 41°42′–42°45′N). The region experiences a temperate continental monsoon climate. From April to June, the average temperature is about 19.7 °C (Fig. 1A), with total precipitation of 227.5 mm (Fig. 1B) and an average humidity of 68.4% (Fig. 1C). The soil is mainly dark brown forest soil, free from saline-alkali stress.

### Saline and alkali stress treatments for the three plants

Following the method for salt stress described by Huang et al. (2020), a mixed neutral salt solution with a NaCl:Na_2_SO_4_ molar ratio of 1:1 was prepared. According to the method for alkali stress outlined by Jia & Li (2021), a mixed alkaline salt solution with a NaHCO_3_:Na_2_CO_3_ molar ratio of 9:1 was prepared. The concentrations for both saline and alkali stress treatments were set at 25, 50, 75, 100, and 125 mmol/L (Fig. 2), with corresponding pH ranges of 7.10–7.30 and 9.19–9.28, respectively. Distilled water was used as the control treatment.

### Germination parameters of the three plants

In the seed germination experiment, plump and uniformly sized seeds were selected, disinfected with 0.1% K_2_MnO_4_ for 10 min, rinsed with distilled water 3–4 times, and blotted dry. The seeds were placed in 10 cm Petri dishes lined with double-layer filter paper. Then, five mL of the corresponding concentration of salt–alkali solution was added to each dish, while the control (CK) received five mL of distilled water. The experiment was conducted in three groups. Group A consisted of 99 Petri dishes, each containing 30 seeds, used to determine germination percentage, germination energy, germination index, and other indicators (Fig. 3A). Group B consisted of 99 Petri dishes, each containing 10 seeds, used to measure radicle length and plumule length (Fig. 3A). Group C consisted of 99 pots, each containing 10 seeds, used to record plant height, biomass, and physiological parameters (Fig. 3B). Each group included three replicates (*i.e.,* three Petri dishes or pots per treatment combination), and the Petri dish or pot was treated as the independent experimental unit. During measurement, the mean value of all subsamples (seeds or seedlings) within each experimental unit was calculated first, and then the unit means were used for subsequent statistical analysis. The Petri dishes were placed in an intelligent light incubator (RTOP-1000D) with day/night temperatures of 25 °C/15 °C, a photoperiod of 12 h light/12 h dark, and a relative humidity maintained at 60%. Starting 24 h after sowing, the number of seeds germinated each day was recorded. Observation was terminated when no new germination was observed for 30 d. According to the standards recommended by the International Seed Testing Association (ISTA, 2018), the germination test duration was 14 d for *C. officinalis* L. and *T. erecta* L., and 21 d for *L. maximum*. Germination energy was calculated on 7 d for *C. officinalis* L. and *T. erecta* L. and on 14 d for *L. maximum*; germination percentage was calculated on 14 d for *C. officinalis* L. and *T. erecta* L. and on 21 d for *L. maximum*. The germination index, vigor index, and relative inhibition rate were also determined.

(1) \begin{eqnarray*}\text{Germination Energy}~(\mathrm{GE},\%)=(\text{Number of seeds germinated by Day 4}/\nonumber\\\displaystyle \text{Total number of tested seeds})\times 100\%\end{eqnarray*}

(2) \begin{eqnarray*}\text{Germination Rate (GR, % )}=(\text{Number of seeds germinated by Day 7}/\nonumber\\\displaystyle \text{Total number of tested seeds})\times 100\%\end{eqnarray*}

(3) \begin{eqnarray*}\text{Germination Index (GI)}=\sum \mathrm{(Gt/Dt)}\end{eqnarray*}

(4) \begin{eqnarray*}\text{Vigor Index (VI)}=\mathrm{GI}\times \mathrm{GL}\end{eqnarray*}

**Figure 1:**
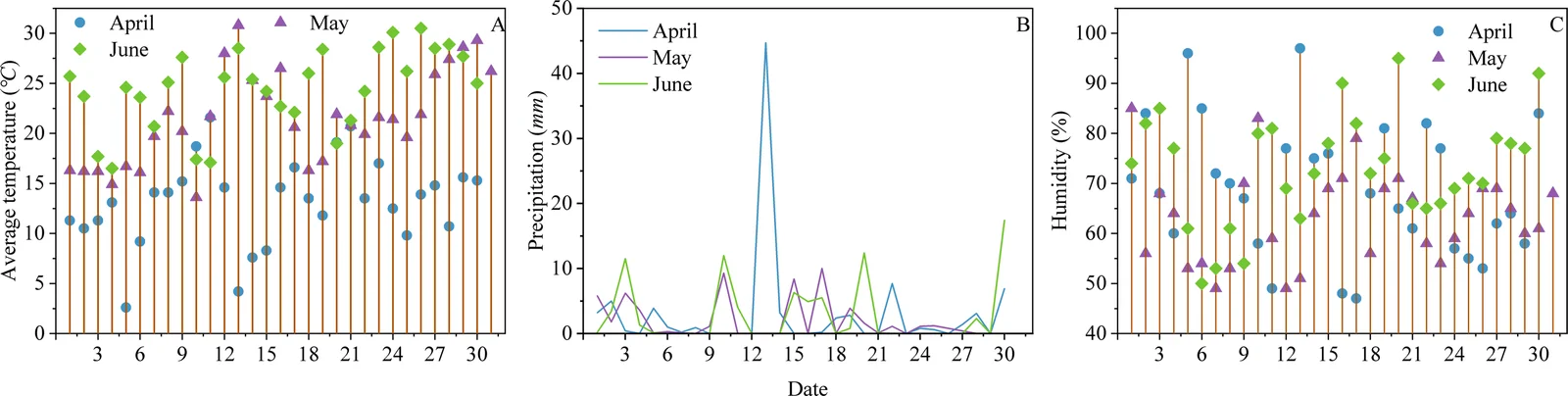
Growing season (April to June) climate conditions in the Songnen Plain region of Jilin Province: (A) average temperature, (B) precipitation, and (C) humidity.

**Figure 2:**
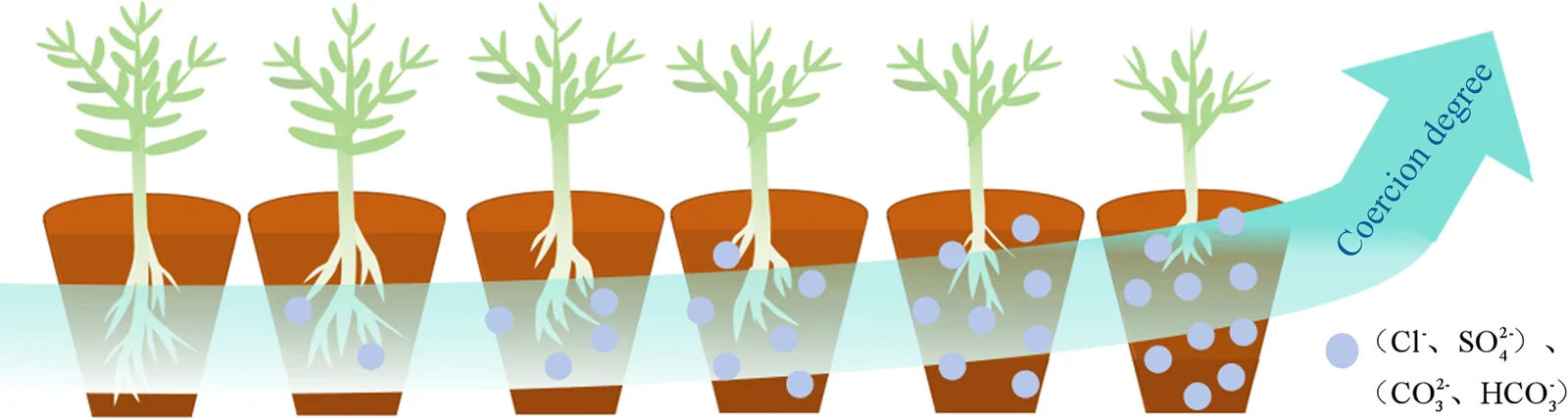
Schematic diagram of the pot experiment treatments for three tested Asteraceae plants under salt and alkali stresses. From left to right, six concentration levels (five graded treatments plus a distilled-water control) were established, with progressively increasing stress intensity for both salt and alkali conditions. These levels respectively constituted asalinity gradientand analkalinity gradient. The blue dots represent the main harmful anions in saline-alkali soil, including Cl^−^ and ${\mathrm{SO}}_{4}^{2-}$ (for salt stress) or ${\mathrm{CO}}_{3}^{2-}$ and $\mathrm{HC}{\mathrm{}O}_{3}^{-}$ (for alkali stress), and the number of dots is positively correlated with the ion concentration. All three tested plants were subjected to the same gradient system to ensure consistency of treatment conditions.

**Figure 3:**
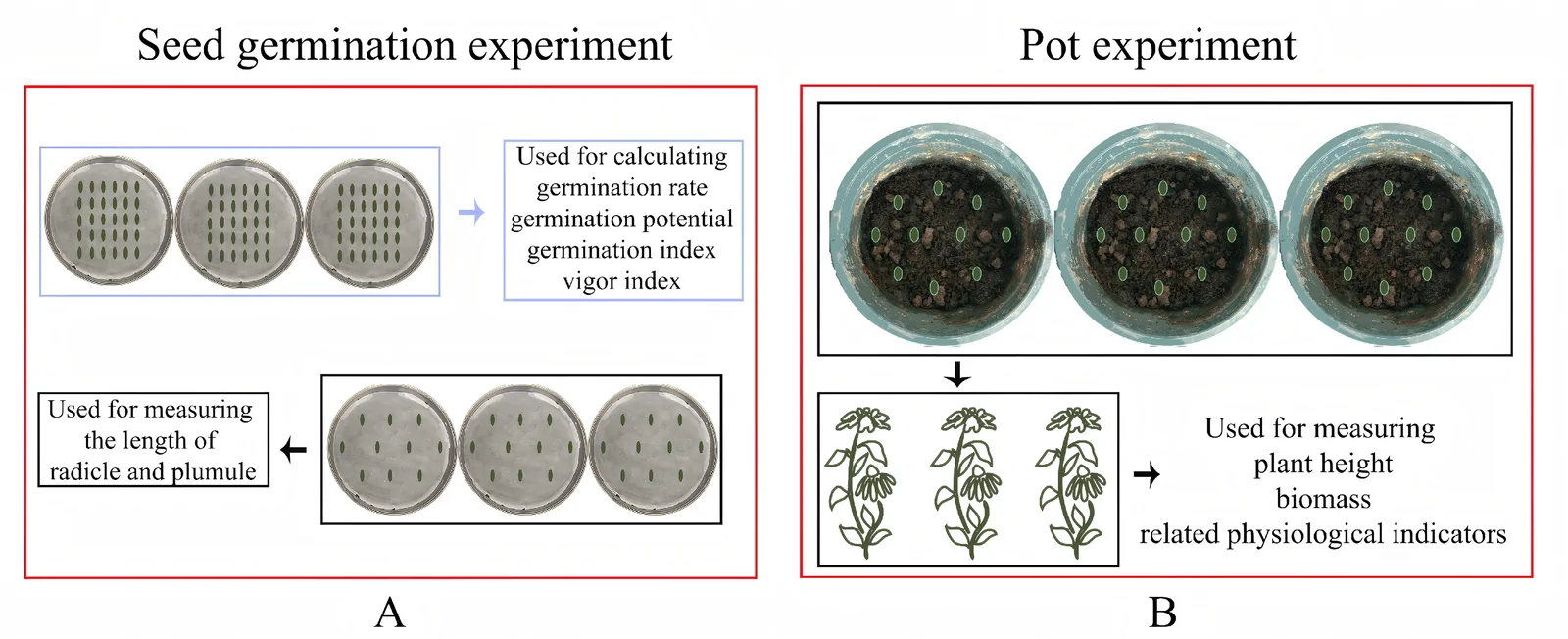
Schematic diagram of experimental design for each treatment group. (A) Seed germination experiment, (B) pot experiment.

where Gt is the daily germination count, and Dt is the corresponding day of germination count.

(5) \begin{eqnarray*}\text{Relative Damage Rate}=(\text{Control Germination Rate}-\text{Stress Treatment}\nonumber\\\displaystyle \text{Germination Rate})/\text{Control Germination Rate}\times 100\%\end{eqnarray*}

(6) \begin{eqnarray*}\text{Relative Root Length (\% )}=(\text{Root Length under Stress Treatment}/\mathrm{Root}\nonumber\\\displaystyle \text{Length under Control})\times 100\%\end{eqnarray*}

(7) \begin{eqnarray*}\text{Relative Shoot Length (\% )}=(\text{Shoot Length under Stress Treatment}/\mathrm{Shoot}\nonumber\\\displaystyle \text{Length under Control})\times 100\%.\end{eqnarray*}

#### Seedling growth parameters of the three plants

In the seedling growth experiment, seeds of three Asteraceae species were sown in pots, and no salt or alkali stress was applied before emergence. On day 7 after emergence, a mixed salt/alkali solution was added. The control treatment received no additional salt or alkali stress. During the seedling growth stage, on day 20 after the stress treatment, three seedlings were randomly selected from each stress treatment. Leaf number and plant height were measured using a vernier caliper, and biomass was weighed using an electronic balance.

#### Determination of physiological parameters in leaves of three plant species

Malondialdehyde (MDA) content, superoxide dismutase (SOD, EC 1.15.1.1) activity, and peroxidase (POD, EC 1.11.1.7) activity were measured using the thiobarbituric acid (TBA) colorimetric method, nitroblue tetrazolium (NBT) reduction method, and guaiacol colorimetric method, respectively, with three technical replicates per sample (Ma et al., 2024).

#### Field experiment

Field experiments were conducted in three areas of the Songnen Plain: Tongyu, Qian’an, and Zhenlai in Jilin Province. Three plots were established in each area, and 50 seeds were uniformly sown in each plot. Two months after sowing, the plants were harvested, and the following parameters were recorded: survival number, leaf number, leaf length, leaf width, root length, plant height, fresh weight, as well as MDA, SOD, and POD activities.

Based on exchangeable sodium percentage (ESP), electrical conductivity (EC), and pH, the soils of the three areas were classified as mild (LS), moderate (MS), and severe (HS) saline-alkali soils (Table 1). All field work was conducted in public green spaces and did not involve private land or restricted areas.

### Data processing

In the statistical analysis, a two-way analysis of variance (two-way ANOVA) was performed separately on the following indicators: germination percentage, germination index, vigor index, leaf length, leaf width, root length, fresh weight, root-shoot ratio, malondialdehyde (MDA), superoxide dismutase (SOD), and peroxidase (POD), with salt type and salt concentration as fixed factors. Prior to analysis, data normality was checked using the Shapiro–Wilk test, and homogeneity of variances was checked using Levene’s test. All data met the assumptions (*p* > 0.05). When significant differences were detected (*p* < 0.05), *post hoc* multiple comparisons were conducted using Tukey’s HSD test, and 95% confidence intervals (CIs) were reported. All data are presented as mean ± standard error (SE).

## Results

### Effects of salt stress and alkali stress on germination characteristics of the three Asteraceae plants

#### Effects of salt stress and alkali stress on germination rate of the three Asteraceae plants

With increasing concentrations of salt stress and alkali stress, the germination rates of both *C. officinalis* L. and *L. maximum* seeds decreased, showing significant reductions compared to the non-stressed control treatment (*p* < 0.05) (Figs. 4A, 4B). In contrast, the germination rate of *T. erecta* L. seeds was highest under a stress concentration of 25 mmol/L for both salt and alkali, and it did not show a significant difference compared with the non-stressed treatment (*p* > 0.05), indicating that low-concentration salt and alkali stress promoted the germination of *T. erecta* L. seeds (Figs. 4A, 4B). Under the non-stressed control condition, the germination rate of *L. maximum* was significantly higher than that of *C. officinalis* L. and *T. erecta* L. (*p* < 0.05). At a stress concentration of 125 mmol/L, the germination rate of *T. erecta* L. became significantly higher than that of both *L. maximum* and *C. officinalis* L. (*p* < 0.05) (Figs. 4A, 4B).

**Table 1:** Soil physicochemical properties of the three experimental sites (Tongyu, Qian’an, and Zhenlai) in the Songnen Plain.

Area	Tongyu	Qian’an	Zhenlai
Salinity level	LS	MS	HS
pH	8.26 ± 0.18	9.14 ± 0.21	9.83 ± 0.26
EC (mS/cm)	1.75 ± 0.13	3.62 ± 0.21	5.17 ± 0.28
ESP (%)	12.45 ± 1.08	24.63 ± 1.72	38.72 ± 2.15
OM (g/kg)	21.35 ± 1.24	14.62 ± 1.07	9.39 ± 0.82
TN (g/kg)	1.12 ± 0.06	0.73 ± 0.05	0.55 ± 0.03
AN (mg/kg)	64.52 ± 3.87	38.64 ± 2.95	23.51 ± 2.16
TP (g/kg)	0.85 ± 0.04	0.61 ± 0.03	0.38 ± 0.02
AP (mg/kg)	13.21 ± 1.03	6.85 ± 0.72	3.49 ± 0.41
TK (g/kg)	23.87 ± 1.22	24.15 ± 1.15	23.92 ± 1.09
AK (mg/kg)	201.4 ± 9.6	143.7 ± 8.4	139.8 ± 7.9

**Notes.**

OM organic matter TN total nitrogen (N) AN available nitrogen TP total phosphorus (P) AP available P TK total potassium (K) AK available K

**Figure 4:**
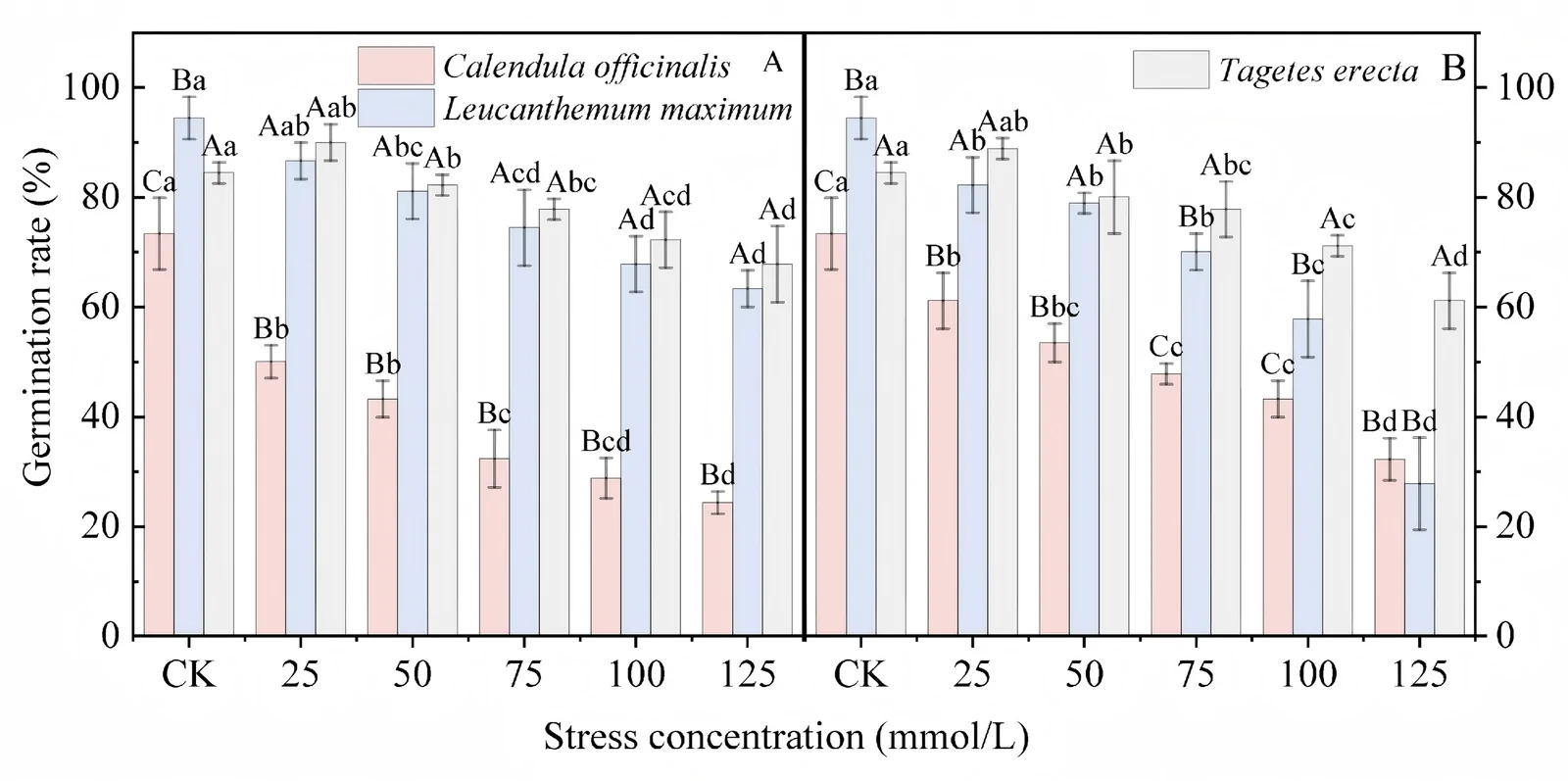
Effects of saline-alkali stress on the germination rates of the three Asteraceae plants. (A) Salt stress, (B) alkali stress. Different uppercase letters indicate significant differences in germination rate among the three plants under the same stress concentration (*p* < 0.05). Different lowercase letters indicate significant differences among different concentration treatments for the same plants (*p* < 0.05).

#### Effects of salt stress and alkali stress on germination energy of the three Asteraceae plants

The germination energy of all three Asteraceae plants reached its lowest value at a stress concentration of 125 mmol/L under both salt and alkali conditions (Figs. 5A, 5B). Under non-stress conditions, no significant difference was observed in germination energy between *T. erecta* L. and *L. maximum* (*p* > 0.05). At a salt stress concentration of 125 mmol/L, there was no significant difference in germination energy between *C. officinalis* L. and *L. maximum* (*p* > 0.05) (Fig. 5A). However, when alkali stress concentration exceeded 75 mmol/L, the germination energy of *C. officinalis* L. became significantly higher than that of *L. maximum* (*p* < 0.05) (Fig. 5B).

**Figure 5:**
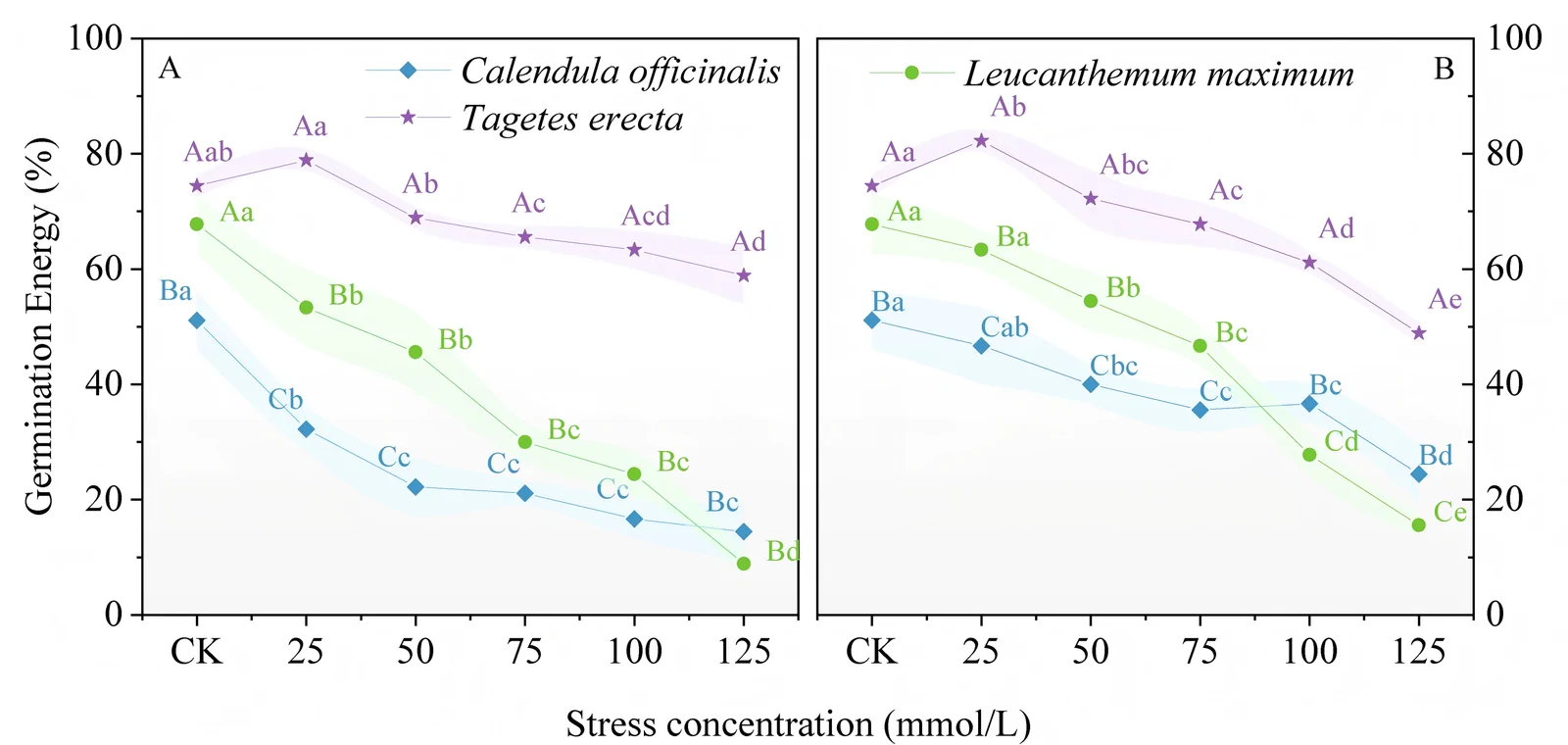
Effects of saline-alkali stress on the germination energy of the three Asteraceae plants. (A) Salt stress, (B) alkali stress. Different uppercase letters indicate significant differences in germination energy among the three plants under the same stress concentration (*p* < 0.05). Different lowercase letters indicate significant differences among different concentration treatments for the same plants (*p* < 0.05).

#### Effects of salt stress and alkali stress on germination index of the three Asteraceae plants

Under non-stress conditions, no significant difference was observed in the germination index between *T. erecta* L. and *L. maximum* seeds (*p* > 0.05) (Figs. 6A, 6B). When the alkali stress concentration exceeded 100 mmol/L, there was no significant difference in the germination index between *L. maximum* and *C. officinalis* L. (*p* > 0.05) (Fig. 6B).

**Figure 6:**
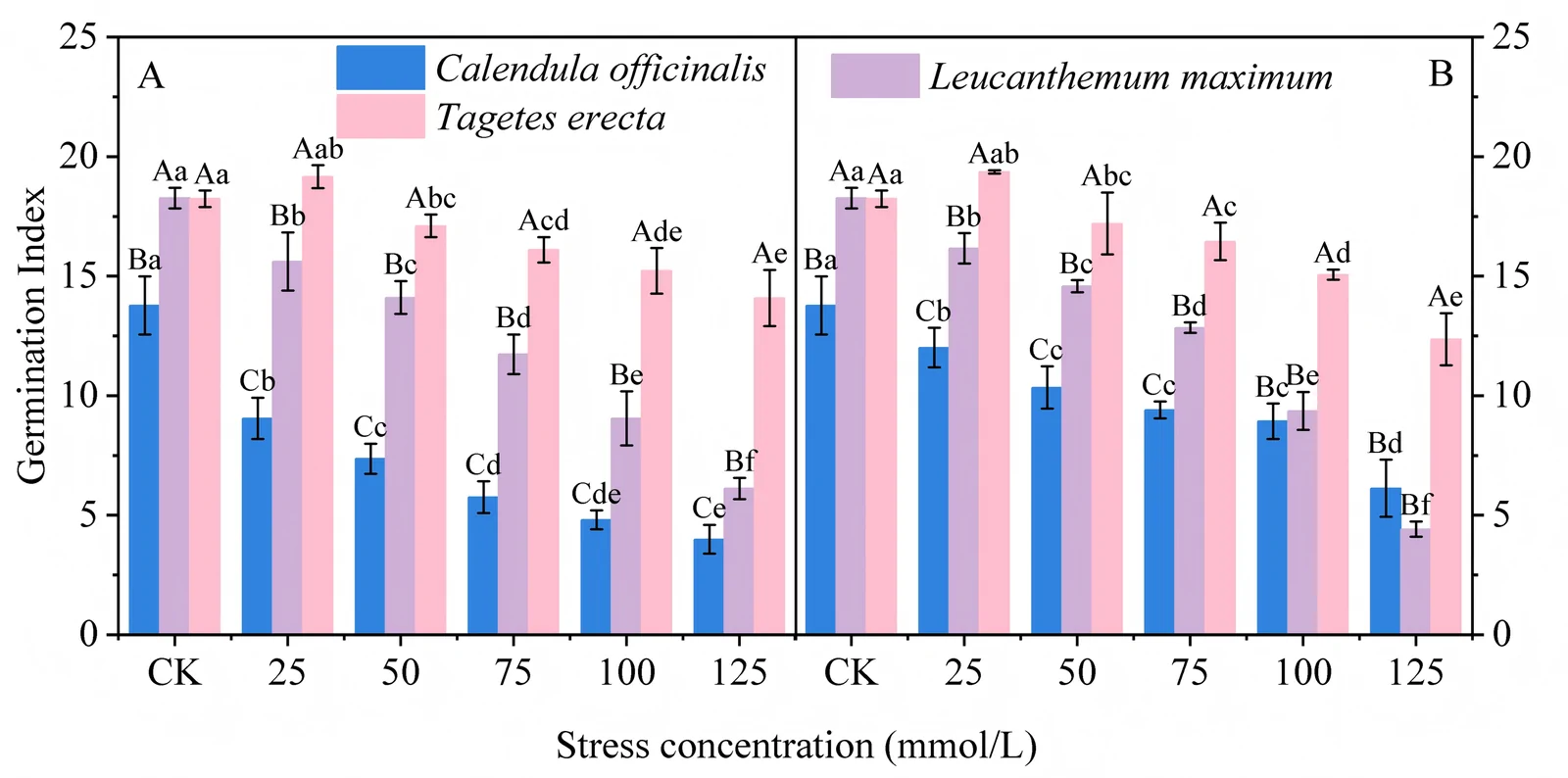
Effects of saline-alkali stress on the germination index of the three Asteraceae plants. (A) Salt stress, (B) alkali stress. Different uppercase letters indicate significant differences in germination index among the three plants under the same stress concentration (*p* < 0.05). Different lowercase letters indicate significant differences among different concentration treatments for the same plants (*p* < 0.05).

#### Effects of salt stress and alkali stress on vigor index of the three Asteraceae plants

Under both salt and alkali stress treatments, the vigor index of *T. erecta* L. seeds was significantly higher than that of *C. officinalis* L. and *L. maximum* (*p* < 0.05) (Figs. 7A, 7B). No significant difference in vigor index was observed between *C. officinalis* L. and *L. maximum* when the salt stress concentration exceeded 75 mmol/L (*p* > 0.05).

**Figure 7:**
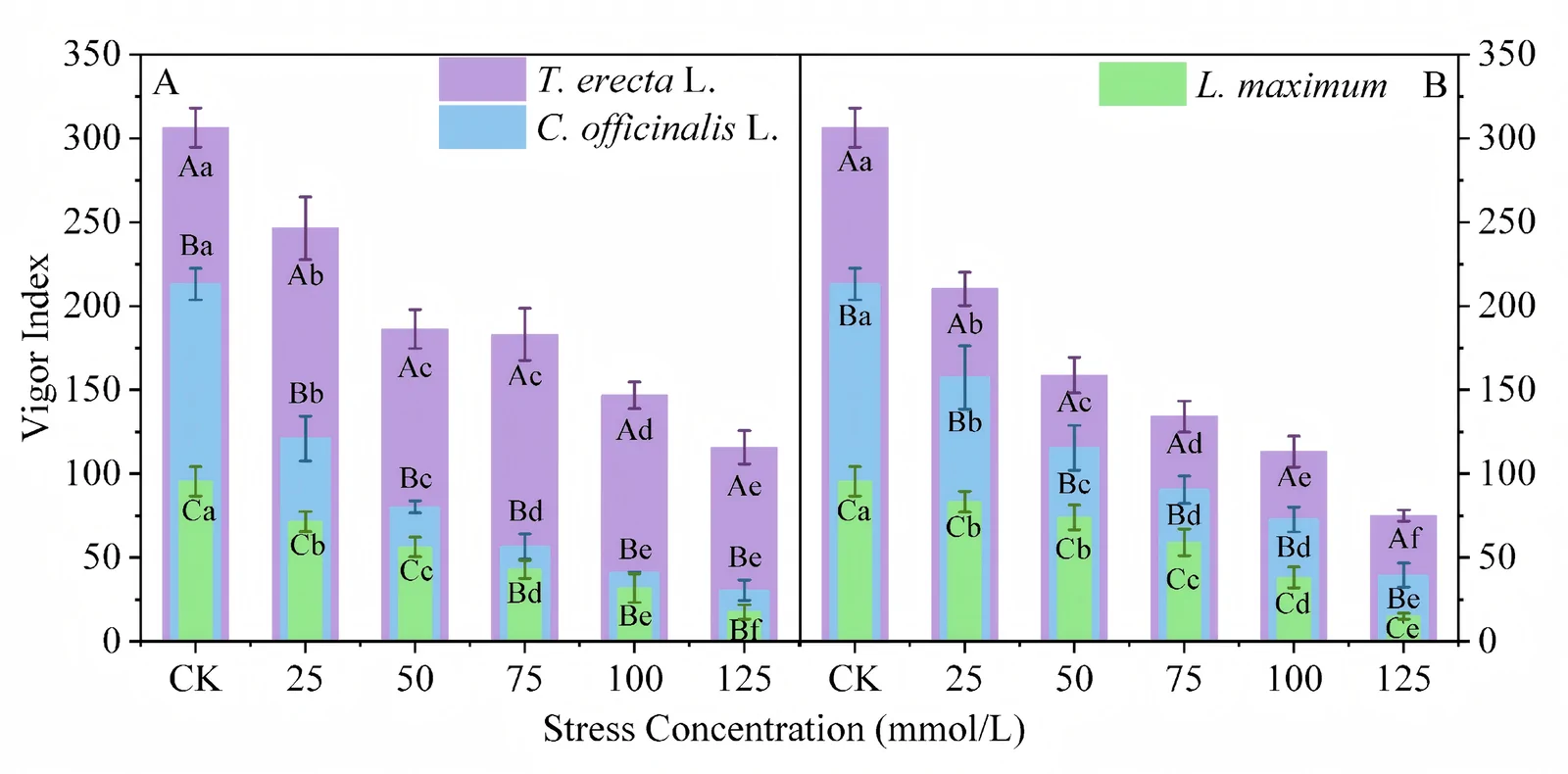
Effects of saline-alkali stress on the vigor index of the three Asteraceae plants. (A) Salt stress, (B) alkali stress. Different uppercase letters indicate significant differences in germination index among the three plant species under the same stress concentration (*p* < 0.05). Different lowercase letters indicate significant differences among different concentration treatments for the same plant species (*p* < 0.05).

#### Effects of salt stress and alkali stress on the relative damage rate of the three Asteraceae plants

With increasing concentrations of both salt and alkali stress, the relative damage rate increased for all three Asteraceae plants. Significant differences were observed in the relative salt injury rate among *C. officinalis* L., *L. maximum*, and *T. erecta* L. (*p* < 0.05). Similarly, the relative alkali injury rate of both *C. officinalis* L. and *L. maximum* showed significant differences compared to that of *T. erecta* L. (*p* < 0.05) (Figs. 8A, 8B).

**Figure 8:**
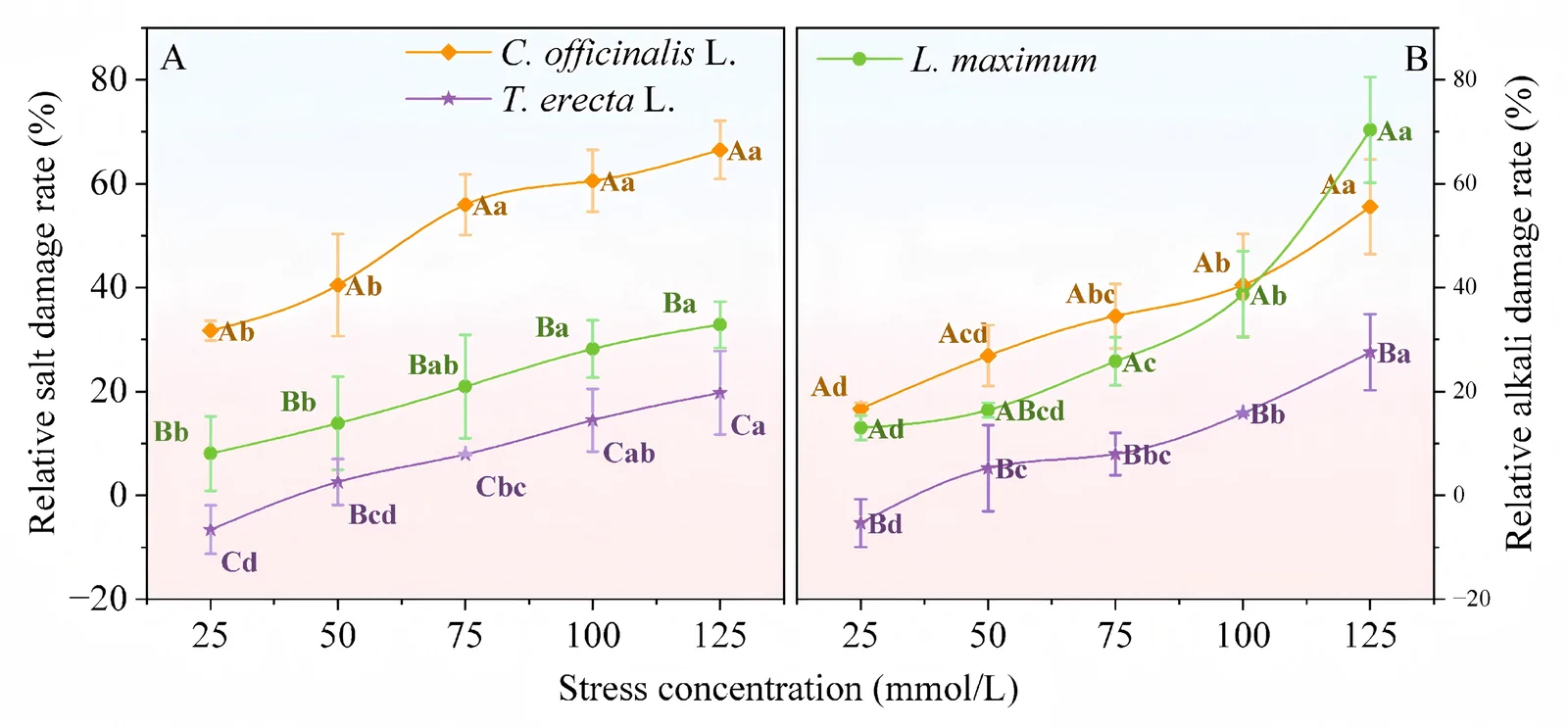
Effects of salt stress and alkali stress on the relative damage rate of the three Asteraceae plants. (A) Salt stress, (B) alkali stress. Different uppercase letters indicate significant differences in germination index among the three plants under the same stress concentration (*p* < 0.05). Different lowercase letters indicate significant differences among different concentration treatments for the same plants (*p* < 0.05).

### Effects of salt stress and alkali stress on seedling growth of the three Asteraceae plants

#### Effects of salt stress and alkali stress on relative shoot length of the three Asteraceae plants

The relative shoot length of all three Asteraceae plants decreased significantly with increasing concentrations of both salt and alkali stress (*p* < 0.05), reaching its lowest value at 125 mmol/L (Figs. 9A, 9B). *T. erecta* L. exhibited the most significant inhibition in shoot length under alkali stress, with a value of 36.65% at 125 mmol/L, which was significantly lower than that of the other two species (*p* < 0.05). *L. maximum* demonstrated a stronger capacity to maintain shoot length under alkali stress, achieving a relative shoot length of 87.76% at 75 mmol/L (Fig. 9B).

**Figure 9:**
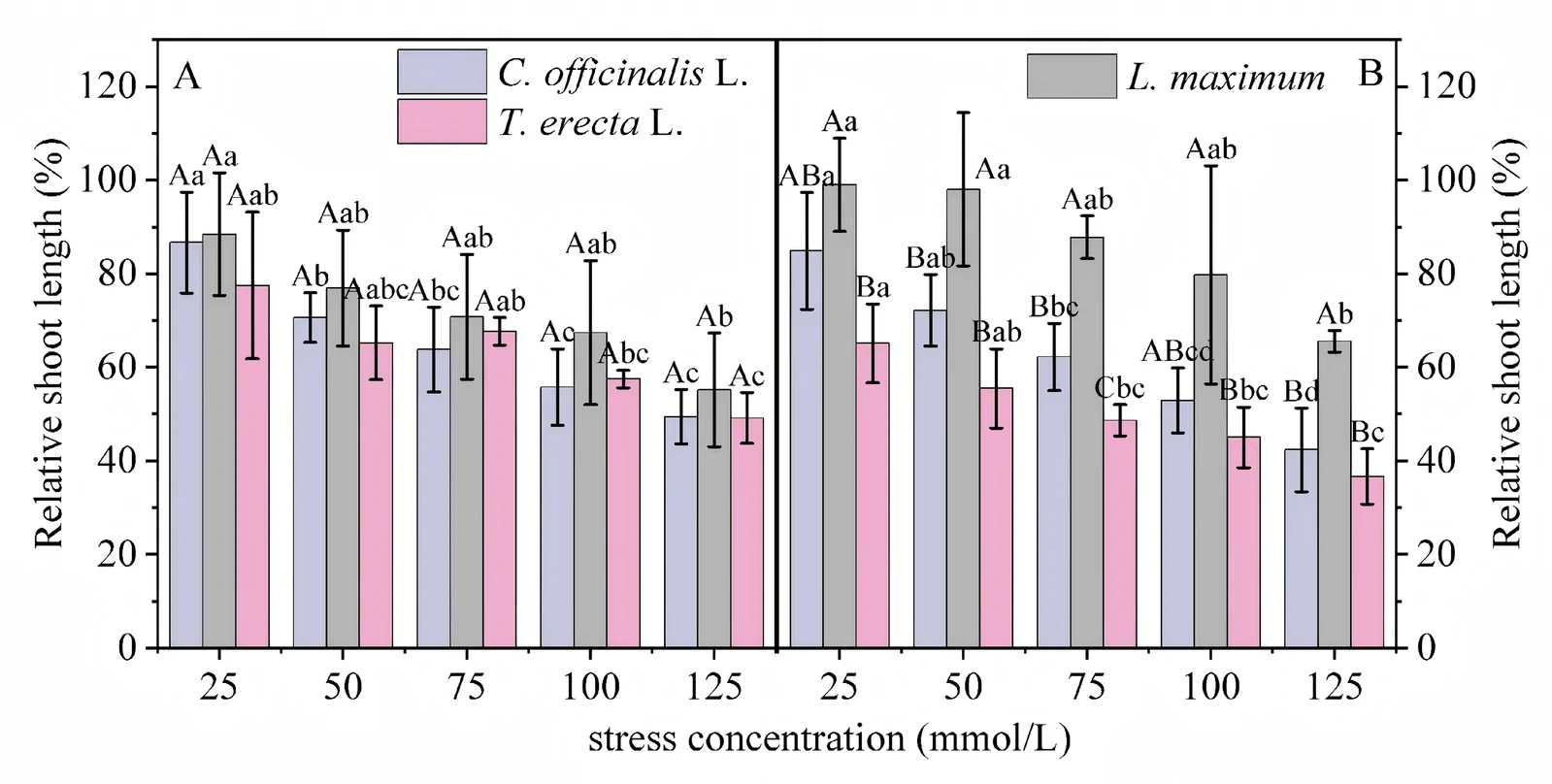
Effects of salt stress and alkali stress on the relative shoot length of seedlings of the three Asteraceae plants. (A) Salt stress, (B) alkali stress. Different uppercase letters indicate significant differences in germination index among the three plants under the same stress concentration (*p* < 0.05). Different lowercase letters indicate significant differences among different concentration treatments for the same plants (*p* < 0.05).

#### Effects of salt stress and alkali stress on relative root length of the three Asteraceae plants

As the concentrations of salt and alkali stress increased, the relative root length of all three Asteraceae plants decreased significantly (*p* < 0.05). *L. maximum* maintained a relatively high relative root length of 83.90% under moderate salt stress (50 mmol/L), indicating relatively strong salt tolerance (Table 2). The inhibitory effect of alkali stress on root length was stronger than that of salt stress. Under the 25 mmol/L condition, the relative root length of *C. officinalis* L. under alkali stress (49.12%) was significantly lower than that under salt stress (86.33%) (Fig. 10).

**Table 2:** Effects of salt stress and alkali stress on the relative root length of the three Asteraceae plants.

**Stress concentration (mmol/L)**	**Relative root length** ** (%)**		
	***C*** ***. o*** ***fficinalis*** ** L.**	***L*** ***.*** ***maximum***	***T*** ***.*** ***e*** ***recta*** ** L.**
Y25	86.33 ± 2.68Aa	77.57 ± 20.72Aab	81.56 ± 4.11Aa
Y50	61.27 ± 3.08Ab	83.90 ± 19.54Aa	69.98 ± 1.71Ab
Y75	52.50 ± 2.35Bc	68.96 ± 5.11Aab	60.00 ± 6.06ABc
Y100	44.91 ± 0.85Bd	56.78 ± 6.56Abc	54.23 ± 4.11ABc
Y125	36.67 ± 1.28Ae	34.66 ± 11.43Ac	31.15 ± 2.42Ad
J25	49.12 ± 4.11Ca	93.17 ± 7.28Aa	67.21 ± 7.63Ba
J50	36.40 ± 3.16Bb	80.78 ± 25.31Aa	59.22 ± 6.00ABa
J75	30.36 ± 2.13Cc	77.19 ± 8.28Aab	48.44 ± 5.76Bb
J100	25.20 ± 2.56Bd	64.15 ± 21.97Aab	37.56 ± 5.11ABc
J125	16.12 ± 2.37Be	44.42 ± 14.90Ab	23.93 ± 5.01Bd

**Notes.**

Y salt stress J alkali stress

Different uppercase letters indicate significant differences in germination index among the three plants under the same stress concentration (*p* < 0.05). Different lowercase letters indicate significant differences among different concentration treatments for the same plants (*p* < 0.05).

**Figure 10:**
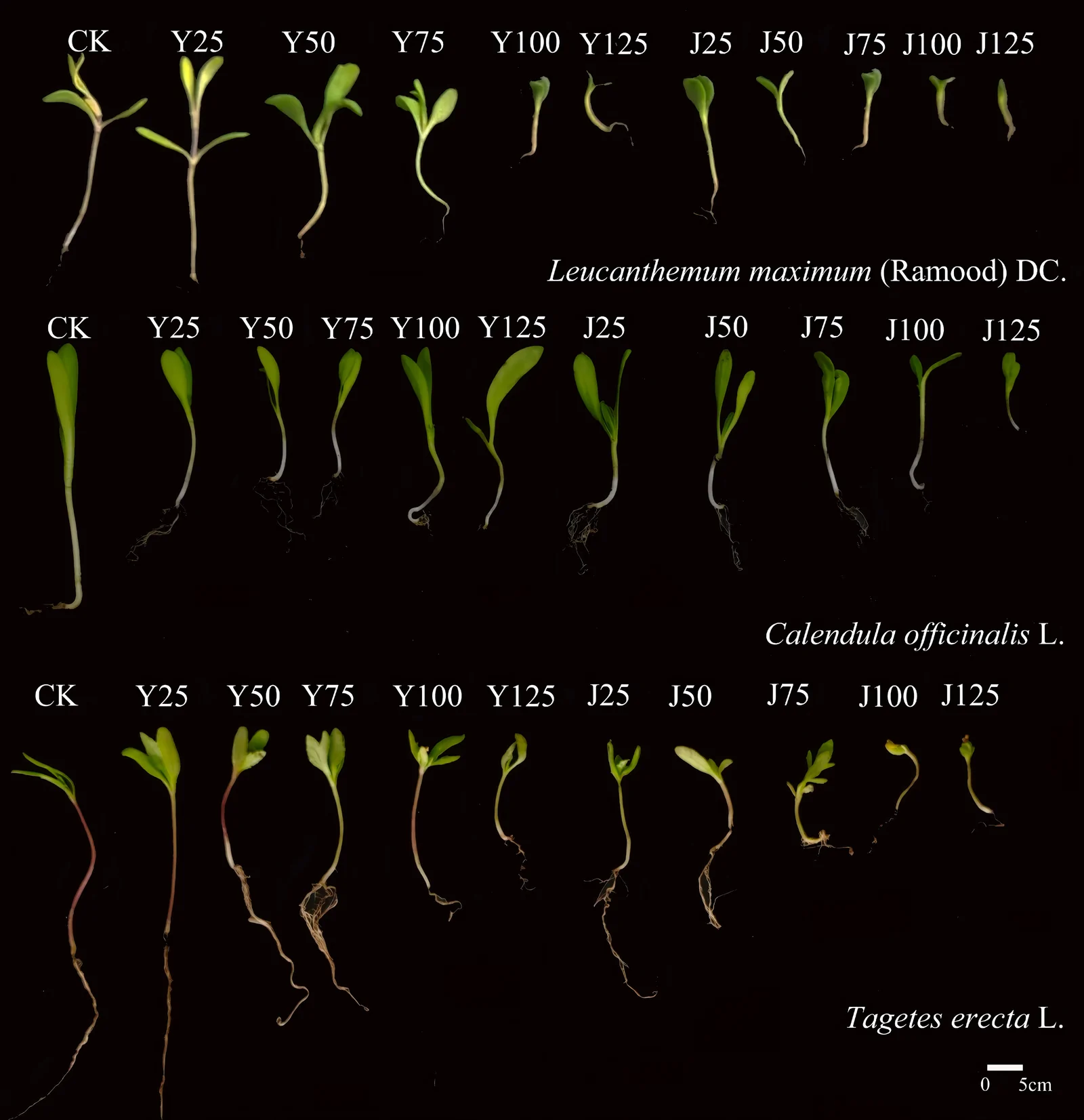
Seedling morphological phenotypes of three Asteraceae species under different saline-alkali stress types and concentrations. CK, blank control; Y25-Y125, neutral salt stress (25–125 mmol/L); J25-J125, alkali stress (same concentrations).

#### Effects of salt and alkali stress on biomass allocation in the three Asteraceae plants

Under both salt and alkali stress treatments, the above-ground and below-ground biomass of all three plants decreased with increasing stress concentrations, accompanied by a decline in the proportion of below-ground biomass (Figs. 11A, 11B, 11C). Under non-stress conditions, there were no significant differences in the proportion of below-ground biomass among the three species (*p* > 0.05) (Figs. 11A, 11B, 11C). At a salt stress concentration of 125 mmol/L, the proportion of below-ground biomass in *T. erecta* L. and *C. officinalis* L. was significantly higher than that in *L. maximum* (*p* < 0.05). Under alkali stress conditions, the proportion of above-ground biomass in *T. erecta* L. was significantly lower than that in *C. officinalis* L. and *L. maximum* (*p* < 0.05).

**Figure 11:**
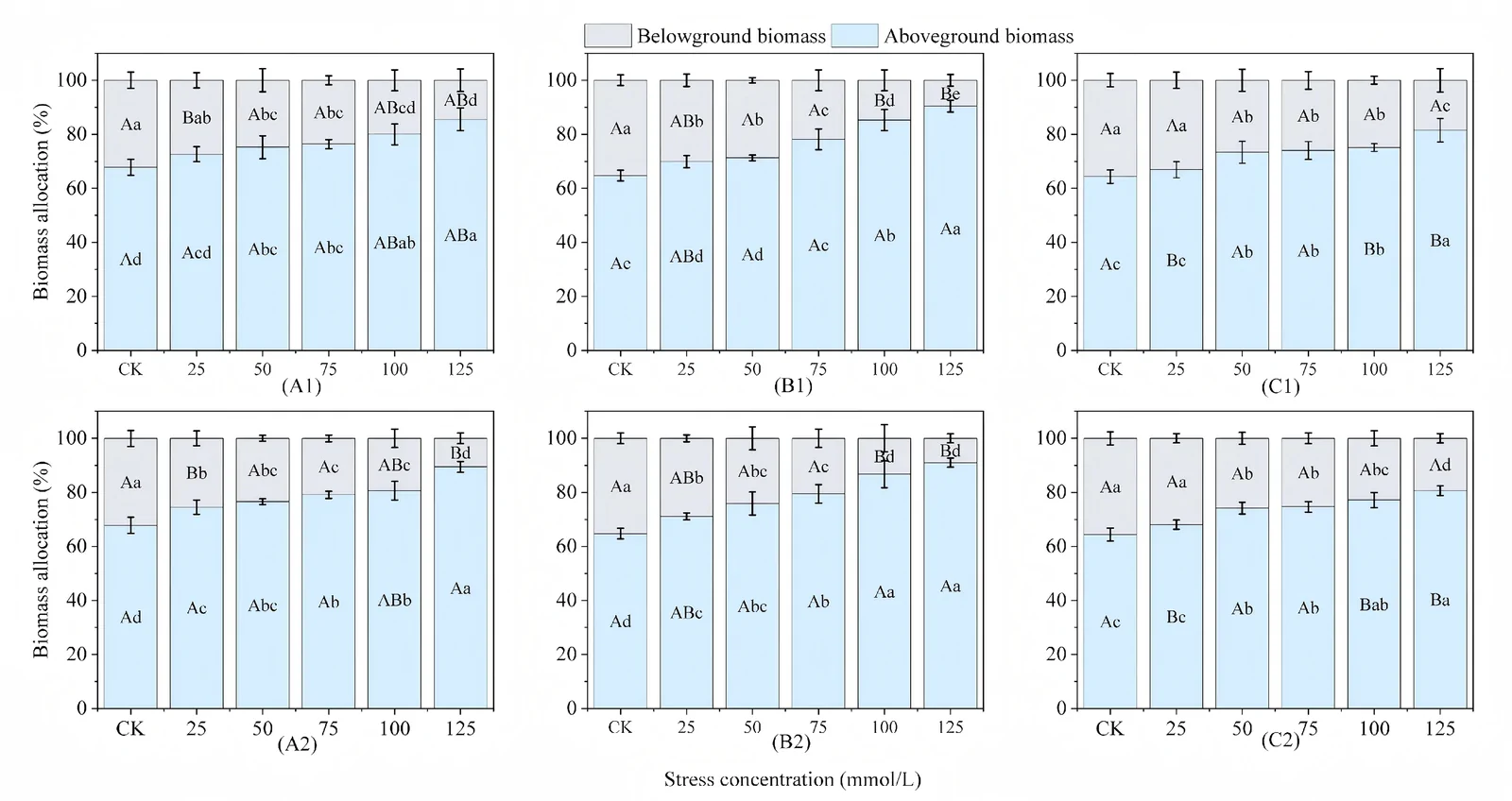
Effects of different saline-alkali stresses on biomass allocation in the three Asteraceae plants. (A) *C. officinalis* L., (B) *L. maximum*, (C) *T. erecta* L., (1) salt stress, (2) alkali stress. Different uppercase letters indicate significant differences in germination index among the three plants under the same stress concentration (*p* < 0.05). Different lowercase letters indicate significant differences among different concentration treatments for the same plants (*p* < 0.05).

#### Effects of salt stress and alkali stress on physiological parameters of three Asteraceae species

With increasing stress concentration, the MDA content of all three species exhibited a continuous increase, and MDA levels under alkali stress were significantly higher than those under salt stress at the same concentration (*p* < 0.05). Both SOD and POD activities showed a trend of initially increasing and then decreasing (Figs. 12A, 12B, 12C). Under the same treatment, the SOD and POD activities of *T. erecta* L. were significantly higher than those of *L. maximum* and *C. officinalis* L. (*p* < 0.05) (Figs. 12B, 12C).

**Figure 12:**
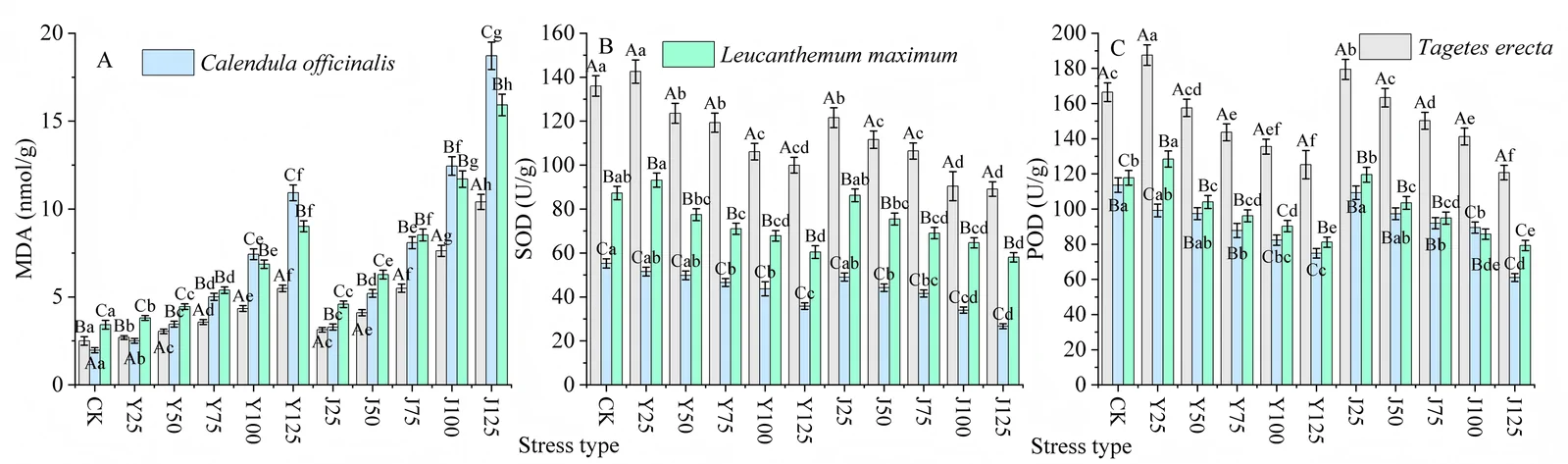
Effects of salt and alkali stresses on physiological parameters of three Asteraceae species. Y, salt stress, J, alkali stress. (A) MDA, (B) SOD, (C) POD. Different uppercase letters indicate significant differences in germination index among the three plants under the same stress concentration (*p* < 0.05). Different lowercase letters indicate significant differences among different concentration treatments for the same plants (*p* < 0.05).

#### Effects of salt and alkali stresses on leaf growth and height of three Asteraceae species

Under all salt and alkali stress treatments, the leaf length, leaf width, and plant height of *C. officinalis* L. were significantly lower than those of the blank control (CK) (*p* < 0.05). In contrast, under 25 mmol/L salt stress, *L. maximum* exhibited significantly greater leaf length, leaf width, and plant height than the CK (*p* < 0.05). For *T. erecta* L., exposure to low-to-medium concentrations of Y25, Y50 (salt stress), and J25 (alkali stress) resulted in significantly increased plant height and leaf width compared to the CK (*p* < 0.05) (Figs. 13A, 13B, 13C).

**Figure 13:**
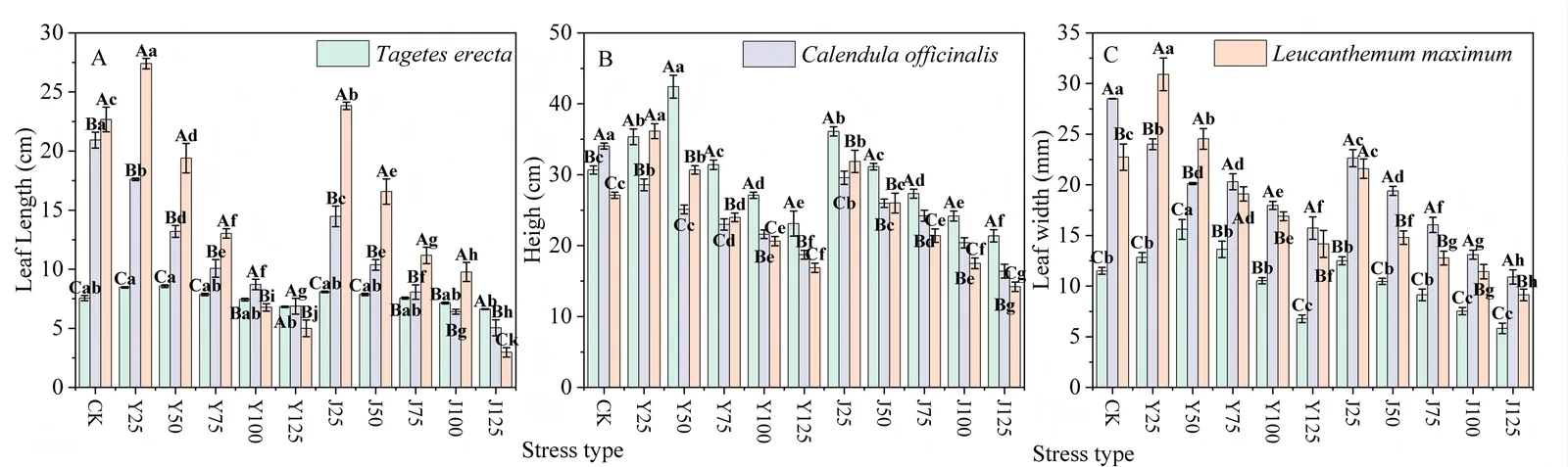
Effects of salt and alkali stresses on leaf growth and plant height of three Asteraceae species. Y, salt stress, J, alkali stress. (A) leaf length, (B) height, (C) leaf width. Different uppercase letters indicate significant differences in germination index among the three plants under the same stress concentration (*p* < 0.05). Different lowercase letters indicate significant differences among different concentration treatments for the same plants (*p* < 0.05).

### Effects of three regions on the traits of three Asteraceae species

With the gradual increase in stress intensity, the survival rate and vegetative growth traits (leaf number, maximum leaf length, maximum leaf width, plant height, root length, fresh weight) of the three plant species showed a continuously and significantly decreasing trend. The content of the lipid peroxidation product MDA exhibited a highly significant continuous increase, while the activities of the core antioxidant enzymes SOD and POD showed a continuously significant decrease (*p* < 0.05) (Figs. 14A, 14J). In the severe saline-alkali region (HS), no significant inhibition of growth traits was observed in *T. erecta* L. (*p* > 0.05), and its survival rate was significantly higher than that of *L. maximum* and *C. officinalis* L. (*p* < 0.05) (Figs. 14A, 14J).

**Figure 14:**
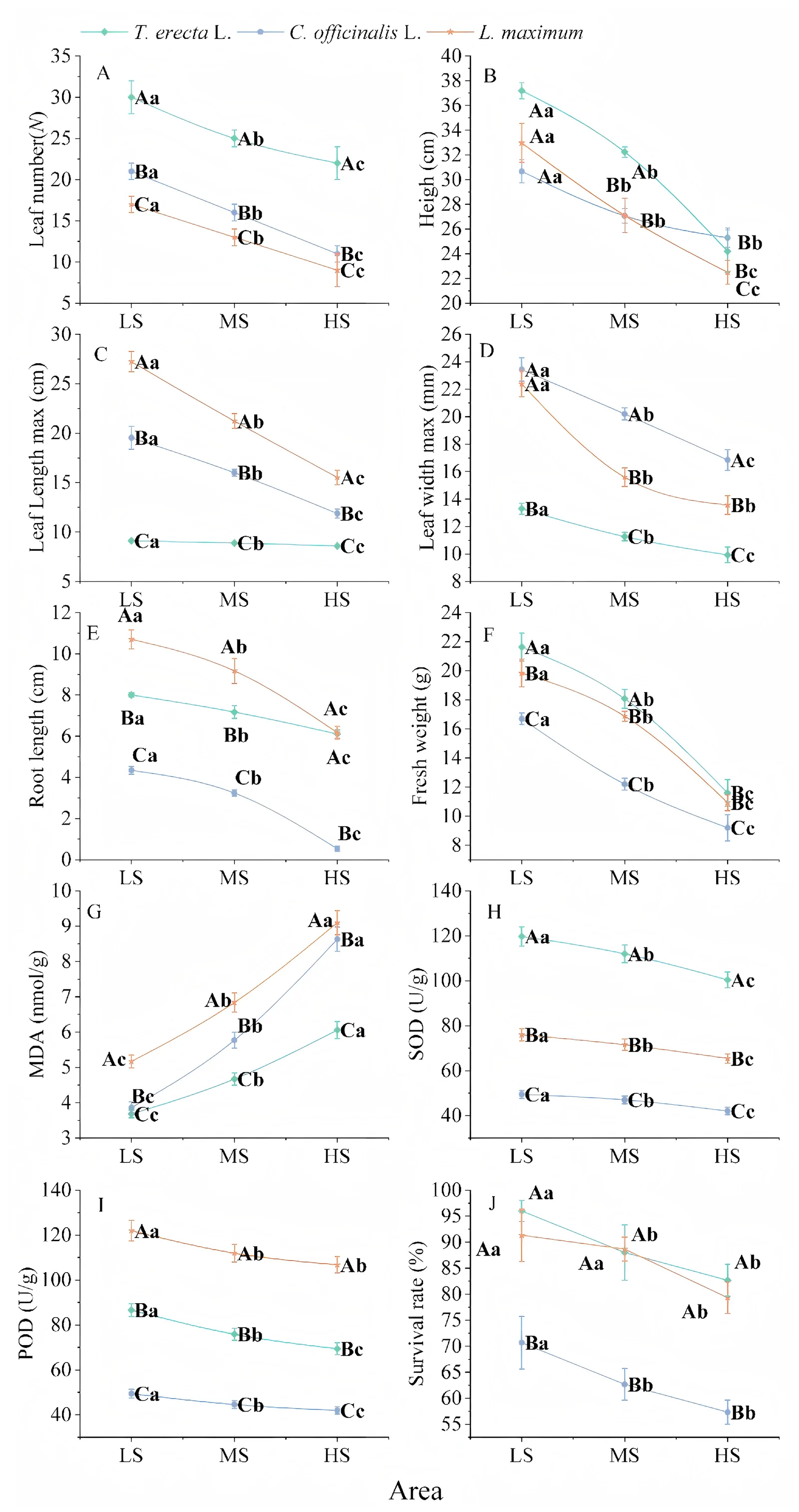
Effects of three regions on the traits of three Asteraceae species. LS, mild saline-alkali soils (Tongyu). MS, moderate saline-alkali soils (Qian’an). HS, severe saline-alkali soils (Zhenlai). (A) Leaf number, (B) height, (C) leaf length max, (D) leaf width max, (E) root length, (F) fresh weight, (G) MDA, (H) SOD, (I) POD, (J) survival rate. Different uppercase letters indicate significant differences in germination index among the three plants under the same stress concentration (*p* < 0.05). Different lowercase letters indicate significant differences among different concentration treatments for the same plants (*p* < 0.05).

### Factorial ANOVA of traits of three Asteraceae under salt stress and alkali stress

For all measured indicators, the main effects of species, stress type, and stress concentration, as well as their two-way interaction effects (species × stress type, species × concentration, stress type × concentration), were all highly significant (*p* < 0.001) (Table 3). This indicates that these three factors are not only core independent regulators of plant responses to saline-alkali stress but also exhibit strong synergistic interactions among them. Except for the root–shoot ratio, the three-way interaction effect (species × stress type × concentration) for the remaining nine indicators was also highly significant (*p* < 0.001), demonstrating that the response patterns of the three plant species to different concentrations of salt and alkali stresses are regulated by a highly significant three-dimensional synergistic mechanism (Table 3).

**Table 3:** Factorial ANOVA of traits of three Asteraceae species under salt stress and alkali stress.

**Pt**	**Sv**	**Sp**	**St**	**C**	**Sp×St**	**Sp×C**	**St×C**	**Sp×St×C**
GR	df	2	1	4	2	8	4	8
	MS	0.9935	0.324	0.539	0.079	0.039	0.0613	0.023
	F	248.38	81	134.75	19.75	9.75	15.31	5.75
	P	<0.01	<0.01	<0.01	<0.01	<0.01	<0.01	<0.01
GI	df	2	1	4	2	8	4	8
	MS	521.29	28.63	289.09	21.43	12.82	4.68	4.05
	F	1,042.58	57.26	578.18	42.86	25.64	9.36	8.1
	P	<0.01	<0.01	<0.01	<0.01	<0.01	<0.01	<0.01
VI	df	2	1	4	2	8	4	8
	MS	212,844.6	10,425.3	95,061.28	6,182.7	5,982.03	3,921.98	2,682.34
	F	638.57	31.28	285.19	18.55	17.95	11.77	8.05
	P	<0.01	<0.01	<0.01	<0.01	<0.01	<0.01	<0.01
LL	df	2	1	4	2	8	4	8
	MS	1,895.62	142.31	1,247.54	17.89	104.27	18.34	10.02
	F	2,487.66	186.72	1,637.43	23.48	136.83	24.07	13.15
	P	<0.01	<0.01	<0.01	<0.01	<0.01	<0.01	<0.01
LW	df	2	1	4	2	8	4	8
	MS	650.28	387.45	874.32	21.46	47.53	29.67	12.35
	F	1,053.92	627.82	1,416.99	34.76	77.02	48.09	20.01
	P	<0.01	<0.01	<0.01	<0.01	<0.01	<0.01	<0.01
RL	df	2	1	5	2	10	5	10
	MS	4,993.04	111.77	1,291.11	113.81	411.22	62.91	44.11
	F	263.21	5.89	68.06	6	21.68	3.32	2.33
	P	<0.01	<0.05	<0.01	<0.01	<0.01	<0.01	<0.05
H	df	2	1	4	2	8	4	8
	MS	320.58	102.47	1,285.79	15.62	58.79	12.35	8.94
	F	417.43	133.52	1,674.27	20.34	76.58	16.09	11.65
	P	<0.001	<0.001	<0.001	<0.001	<0.001	<0.01	<0.01
RSR	df	2	1	4	2	8	4	8
	MS	0.2105	0.182	0.441	0.0155	0.024	0.0155	0.0054
	F	66.41	57.41	139.12	4.89	7.57	4.89	1.7
	P	<0.01	<0.01	<0.01	<0.05	<0.01	<0.01	0.118
MDA	df	2	1	4	2	8	4	8
	MS	72.399	225.967	203.938	3.557	19.854	24.714	8.376
	F	571.42	1,783.47	1,609.61	28.07	156.7	195.06	66.11
	P	<0.01	<0.01	<0.01	<0.01	<0.01	<0.01	<0.01
SOD	df	2	1	4	2	8	4	8
	MS	28,791.11	1,170.99	4,190.02	101.91	210.01	100.01	30.12
	F	3,701.43	150.45	538.72	13.09	26.99	12.85	3.87
	P	<0.01	<0.01	<0.01	<0.01	<0.01	<0.01	<0.01
POD	df	2	1	4	2	8	4	8
	MS	37,001.24	1,008.11	5,940.02	102.34	245.67	110.23	45.78
	F	2,143.31	58.34	343.82	5.92	14.23	6.38	2.65
	P	<0.01	<0.01	<0.01	<0.01	<0.01	<0.01	<0.05

**Notes.**

Pt Plant traits Sv Source of variation Sp species St stress type C concentration GR germination rate GI germination index VI vigor index LL leaf length LW leaf width RL root length H height

## Discussion

### Effects of salt and alkali stress on seed germination of the three Asteraceae plants

The seed germination stage is the most sensitive period for plants under saline-alkali stress. Germination performance reflects their saline-alkali tolerance and is closely related to successful establishment under adverse conditions (Su et al., 2022). High-vigor seeds can maintain better growth performance even in stressful environments (Koch et al., 2021; Izadi-Darbandi & Gresshoff, 2021). Manono (2026) reported that high salinity levels reduce seed germination percentage and prolong germination time. This study found that the germination energy, germination rate, germination index, and vigor index of *C. officinalis* L. and *L. maximum* gradually decreased with increasing concentrations of both salt and alkali stress, indicating that high-concentration saline and alkali stress inhibit the germination of their seeds. This aligns with the findings of Yu et al. (2024) in their study on the saline-alkali tolerance of oregano seeds during germination. Alkaline stress treatment can break seed dormancy (Fan et al., 2021). Low-concentration saline-alkali stress promotes the germination of many plants. In this study, *T. erecta* L. showed promoted germination only under low-concentration (25 mmol/L) stress, which is consistent with the “low-promotion and high-inhibition” phenomenon observed in *Lycium ruthenicum* and seed lotus (Nie et al., 2021; Xu, 2022). This research reveals the differential responses of the three plants to saline-alkali stress. *T. erecta* L. may possess a germination advantage in lightly saline-alkali soils. These findings provide crucial threshold data for selecting plant materials suitable for different degrees of salinization and for developing localized land management strategies, thereby promoting precision agriculture and sustainable ecological restoration practices based on plant tolerance levels.

Saline-alkali stress commonly inhibits seed germination rates, delays germination time, and suppresses plumule growth (Mouhssine et al., 2021; Atta et al., 2023). In this study, alkali stress exerted a stronger inhibitory effect on seed germination of the three Asteraceae plants compared to salt stress. Under high concentrations (≥75 mmol/L), the germination rate and germination index of *C. officinalis* L. and *L. maximum* decreased markedly. The inhibitory effect of alkali stress on seed germination was generally greater than that of salt stress for all three plants, indicating that alkaline salts have a stronger suppressive impact than neutral salts on their seed germination. Compared with salt stress, an alkaline environment also reduces germination rate and germination index more severely in *Onobrychis viciifolia*, *Brassica napus*, and *Oryza sativa* L. (Li et al., 2021; Wang et al., 2022; Yang, Zhang & Li, 2023). Although *L. maximum* exhibited the highest germination rate under non-stress conditions, it showed the greatest decline as stress intensity increased, suggesting a relatively narrow range of adaptation to saline-alkali environments. The differences in the responses of these three Asteraceae plants to saline-alkali stress during the germination stage provide a direct and rapid evaluation criterion for selecting suitable Asteraceae plant materials characterized by high germination rates and strong root system growth, to be applied in agricultural production and ecological restoration of saline-alkali lands.

### Effects of salt and alkali stress on seedling growth of the three Asteraceae plants

The seedling growth stage is a critical period for plants to adapt to saline-alkali environments. Relative root length and relative shoot length serve as important morphological indicators for evaluating the saline-alkali tolerance of seedlings, reflecting adaptive changes in biomass allocation and organ growth under stress conditions (Rekha et al., 2022; Cao et al., 2023; Zhuang et al., 2023). As stress concentration increased, both relative root length and relative shoot length of the three Asteraceae plants decreased significantly, indicating a pronounced inhibitory effect of stress on seedling morphogenesis. Compared to salt stress, alkali stress exerted a stronger inhibitory effect on the root length of *C. officinalis* L., suggesting its higher sensitivity to alkaline environments. Alkaline stress has a more pronounced inhibitory effect on root and shoot elongation in blueberry (Guy et al., 2023). *L. maximum* maintained relatively high relative root length under salt stress, demonstrating stronger salt tolerance, whereas *T. erecta* L. showed the most significant inhibition in shoot length under alkali stress, indicating a weaker adaptability of its above-ground parts to alkaline conditions. Different species may allocate tolerance costs by regulating the growth of different organs, forming diverse adaptation strategies to saline-alkali stress (Song et al., 2022; Ludwiczak et al., 2023). The differences in growth responses reflect the divergence in saline-alkali adaptation strategies among species. Analyzing the differential responses of radicles and plumules to stress reveals the morphological adaptation strategies and survival trade-offs of plants in saline-alkali environments, providing direction for stress-tolerant breeding.

The seedling stage is a critical period for plants to activate antioxidant defense systems under saline-alkali stress. Membrane stability and enzyme activity reflect the capacity to mitigate oxidative damage and are closely linked to survival under adverse conditions (Ma et al., 2024). This study found that MDA content increased continuously with rising stress concentrations in all three Asteraceae species, while SOD and POD activities exhibited a typical “low-promotion and high-inhibition” trend. Notably, alkali stress induced significantly higher MDA accumulation and stronger suppression of enzyme activities compared to salt stress at equivalent concentrations. Compared with salt stress, oxidative damage in *Caragana liouana* is more severe under alkali stress (Wu et al., 2023; Ma et al., 2024). These findings provide a physiological basis for targeted species selection and precision management in saline-alkali soil vegetation restoration.

Saline-alkali stress significantly inhibits plant growth as a general rule, and multiple physiological indicators often show a coordinated decline, reflecting the typical characteristic of overall metabolic impairment in plants under adversity (Jahan et al., 2021; Guo et al., 2022; Zhuang et al., 2023). In this study, under saline-alkali stress, a positive correlation was observed between stress concentration and the relative damage rate, while various physiological indicators exhibited coordinated growth. This synergistic response pattern aligns with findings in other studies, such as those on rice, and represents a conservative strategy for plants coping with saline-alkali stress (Shaban et al., 2023). We speculate that the contrasting resource allocation patterns observed here are fundamentally linked to life history strategy—with *T. erecta* L. (annual) potentially prioritizing rapid germination to exploit transient opportunities, *L. maximum* (perennial) likely favoring root investment for long-term persistence, and *C. officinalis* L. (biennial) exhibiting an intermediate response—although direct experimental evidence for this hypothesis is currently lacking and warrants future investigation (McMorris & Ledder, 2021; Luo, Zhang & Fang, 2021). Similar to the pattern observed in *T. erecta* L. in the current study, previous work on annual species has noted a strategy favoring rapid germination even under mild stress to exploit ephemeral resource windows. Conversely, the stable root growth maintenance observed in *L. maximum* under salt stress aligns with traits reported in other perennial species, which prioritize long-term survival over immediate above-ground expansion. This research can guide the selection of “pioneer species” in saline-alkali land vegetation restoration, prioritizing plants with less inhibited germination and strong seedling establishment capacity to improve the success rate of plant establishment.

## Conclusions

This study systematically compared the responses of three Asteraceae species to salt stress and alkali stress, revealing significant interspecific differences in tolerance thresholds and adaptive strategies. In this study, the alkaline salt mixture exhibited stronger inhibitory effects than the neutral salt mixture. Based on short-term field performance observations, the following preliminary screening results can be drawn: *T. erecta* L. may be considered as a candidate species for rapid establishment in light to moderately saline-alkali lands; *C. officinalis* L. shows potential for short-term coverage in low-salinity areas; and *L. maximum*, owing to its relatively stable root system and salt tolerance, may be more suitable for medium- to long-term ecological restoration trials. This study provides preliminary insights into interspecific differences in saline-alkali tolerance among the three species under the specific experimental conditions, offering a reference basis for species selection and scientific allocation in ecological restoration and landscape greening in saline-alkali regions of China. The findings have potential practical value for improving vegetation coverage and landscape ecological benefits in saline-alkali lands, but still need to be confirmed through further field validation over longer time scales.

##  Supplemental Information

10.7717/peerj.21814/supp-1 Supplemental Information 1 Number of sprouts, sprout length, root length, biomass

## Additional Information and Declarations

### Data Availability

The following information was supplied regarding data availability: The raw data are available in the Supplemental File.

## References

[ref-1] Atta K, Mondal S, Gorai S, Singh AP, Kumari A, Ghosh T, Roy A, Hembram S, Gaikwad DJ, Mondal S, Bhattacharya S, Jha UC, Jespersen D (2023). Impacts of salinity stress on crop plants: improving salt tolerance through genetic and molecular dissection. Frontiers in Plant Science.

[ref-2] Cao YX, Wang J, Zhao SQ, Fang QX, Ruan JW, Li SL, Liu TX, Qi YX, Zhang L, Zhang XM, Meng FL (2023). Overexpression of the aldehyde dehydrogenase *AhALDH3H1* from *Arachis hypogaea* in soybean increases saline-alkali stress tolerance. Frontiers in Plant Science.

[ref-3] Corwin DL, Yemoto K (2020). Salinity: electrical conductivity and total dissolved solids. Soil Science Society of America and American Society of Agronomy.

[ref-4] Dong F, Wang YN, Zhu J, Ma L, Qi Y, Wang CP, Wen C, Zhang JH, Lyu XH (2021). Effects of NaCl stress on seed germination of five compositae species. Shandong Agricultural Sciences.

[ref-5] Dos Santos TB, Ribas AF, De Souza SGH, Budzinski IGF, Domingues DS (2022). Physiological responses to drought, salinity, and heat stress in plants: a review. Stresses.

[ref-6] Fan Q, Ren YJ, Guo XY, Sun HS, Zhao ML (2021). Contrastive study on seed initiation of helianthus tuberosus by different treatments. Seed.

[ref-7] Fang S, Hou X, Liang X (2021). Response mechanisms of plants under saline-alkali stress. Frontiers in Plant Science.

[ref-8] Guo JX, Lu XY, Tao YF, Guo HJ, Min W (2022). Comparative ionomics and metabolic responses and adaptive strategies of cotton to salt and alkali stress. Frontiers in Plant Science.

[ref-9] Guy T, Zeng QL, Dagan E, Zilkah S, Bar-Tal A, Dai N (2023). Combined effects of alkaline pH and high Ca concentration on root morphology, cell-wall polysaccharide concentrations and blueberry plant performance. Frontiers in Agronomy.

[ref-10] Huang Y, Guo M, Zhang HR, Zhou Y, Li HM, Gao ZM, Wang PP (2020). Effects of salt stress on seed germination and seedling growth of carnation. Acta Prataculturae Sinica.

[ref-11] International Seed Testing Association (ISTA) (2018). International Rules for Seed Testing. Bassersdorf, Switzerland. https://www.seedtest.org/en/publications/international-rules-seed-testing.html.

[ref-12] Izadi-Darbandi A, Gresshoff PM (2021). Role of hydroxymethylglutaryl-coenzyme A (HMG-CoA) reductase 1 in nodule development of soybean. Journal of Plant Physiology.

[ref-13] Jahan B, Rasheed F, Sehar Z, Fatma M, Iqbal N, Masood A, Anjum NA, Khan NA (2021). Coordinated role of nitric oxide, ethylene, nitrogen, and sulfur in plant salt stress tolerance. Stresses.

[ref-14] Jia XF, Li B (2021). Effects of NaHCO_3_+Na_2_CO_3_ combined saline alkali stress on physiological metabolism of alfalfa seedlings. Heilongjiang Animal Science and Veterinary Medicine.

[ref-15] Jing H, Sun JQ, Li YQ, Jiang LH, He QY, Li J (2024). Analysis of the ecological value of pioneer plants in the yellow river basin. Asian Journal of Agricultural and Horticultural Research.

[ref-16] Jing MB, Wang JC, Zhang W, Zhou LH, Zhang SP (2022). Comparison of phytoremediation effects of *Medicago sativa* and *Coreopsis basalis* on crude-oil-contaminated soil in eastern Gansu Province. Arid Land Geography.

[ref-17] Koch F, Rolim JM, Martinazzo EG, Pedó T, Villela FA, Aumonde TZ (2021). Growth and dry matter partition wheat plants in response to seed vigor and water restriction. Journal of Seed Science.

[ref-18] Kong XM, Zhang Y, Wang ZY, Bao ST, Feng YF, Wang JQ, Yu ZJ, Long F, Xiao ZJ, Hao YN, Gao XT, Li YF, Ding Y, Wang JY, Xu CYLeiTY, Wang JP (2023). Two-step model of paleohexaploidy, ancestral genome reshuffling and plasticity of heat shock response in Asteraceae. Horticulture Research.

[ref-19] Li ZL, Ma YH, Sun W, Ding PJ, Bu YF, Qi YH, Shi TR, Jia CC, Lei BL, Ma C (2025). The *N6*-methyladenosine reader ECT1 regulates seed germination *via* gibberellic acid- and phytochrome B-mediated signaling. Plant Physiology.

[ref-20] Li SJ, Zhu YH, White JF, Wei M, Wu GQ (2021). Two sainfoin (*Onobrychis viciifolia* Scop.) cultivars differ in their responses to neutral and saline-alkali stress during seed germination and early seedling growth. Applied Ecology and Environmental Research.

[ref-21] Liu C, Jiang X, Yuan Z (2024). Plant responses and adaptations to salt stress: a review. Horticulturae.

[ref-22] Liu JH, Wang B, Pan WZ, Zhu YL, Zi WL, Wu HB, Fang GH, Zhao YZ, Wang QH, Zhang RQ, Wang YM (2021). Effects of marigold straw organicfertilizer + different biological agents mixed application on root-knot nematode control, growth of tobacco. Guizhou Agricultural Sciences.

[ref-23] Liu XH, Zhai YT (2025). Research progress on salt tolerance of Asteraceae plants. Contemporary Horticulture.

[ref-24] Ludwiczak A, Ciarkowska A, Dehnavi AR, Cárdenas-Pérez S, Piernik A (2023). Growth stage-organ- and time-dependent salt tolerance of halophyte *Tripolium pannonicum* (Jacq.) Dobrocz. Life.

[ref-25] Luo WL, Zhang B, Fang QE (2021). Advances in population ecology and reproductive biology of *Stellera chamaejasme*. Journal of Zhejiang A & F University.

[ref-26] Ma ZJ, Zhang J, Wei LL, Ma F, Xu TT (2024). Physiological and growth responses of *Caragana liouana* seedlings to salt-alkali stress. Pratacultural Science.

[ref-27] Manono BO (2026). Effects of salinity on seed germination: mechanisms, impacts, and mitigation strategies. Seeds.

[ref-28] McMorris D, Ledder G (2021). Resource allocation in annual plants. BioRxiv.

[ref-29] Mouhssine F, Ech-Cheddadi S, Ouhaddach M, Goumi YE, Yacoubi HE, Rochdi A (2021). Selection of tolerant wheat varieties during the germination phase. Frontiers in Plant Science.

[ref-30] Nie BL, Wu LM, Nigmat R, Wang DN, Lv HY (2021). Alleviating effects of exogenous melatonine on seed germination and seedling growth of *Lycium ruthenicum* under complex salt-alkali stress. Acta Botanica Boreali-Occidentalia Sinica.

[ref-31] Ondrasek G, Rathod S, Manohara KK, Gireesh C, Anantha MS, Sakhare AS, Parmar B, Yadav BK, Bandumula N, Raihan F, Zielińska-Chmielewska A, Meriño Gergichevich C, Reyes-Díaz M, Khan A, Panfilova O, Seguel Fuentealba A, Romero SM, Nabil B, Wan C, Shepherd J, Horvatinec J (2022). Salt stress in plants and mitigation approaches. Plants.

[ref-32] Peng S, Jin YZ, Chen YQ, Wu CM, Wang YJ, Wang XW, Jin QJ, Xu YC (2022). Growth response, enrichment effect, and physiological response of different garden plants under combined stress of polycyclic aromatic hydrocarbons and heavy metals. Coatings.

[ref-33] Qu FC, Chen WP, Wang JJ (2025). Discussion on engineering treatment model for Dryland of saline-sodic soil in the Songnen Plain based on cause analysis. Acta Ecologica Sinica.

[ref-34] Rekha G, Abhilash KV, Gokulan CG, Koushik MBVN, Laxmi PB, Kulkarni S, Aleena D, Harika G, Hajira SK, Pranathi K, Punniakoti E, Kale RR, Dilip KT, Ayyappa D, Anila M, Sinha P, Manohara KK, Padmavathi G, Subba RLV, Laha GS, Prasad MSS, Fiyaz RA, Suneetha K, Balachandran SM, Patel HK, Sonti RV, Senguttuvel P, Sundaram RM (2022). DRR Dhan 58, a seedling stage salinity tolerant NIL of improved Samba Mahsuri shows superior performance in multi-location trials. Rice.

[ref-35] Shaban AS, Safhi FA, Fakhr MA, Pruthi R, Abozahra MS, El-Tahan AM, Subudhi PK (2023). Comparison of the morpho-physiological and molecular responses to salinity and alkalinity stresses in rice. Plants.

[ref-36] Shahrokhnia H, Wu LS (2021). Saleach: a new web-based soil salinity leaching model for improved irrigation management. Agricultural Water Management.

[ref-37] Song X, Yang N, Su Y, Lu X, Liu J, Liu Y, Zhang Z, Tang Z (2022). Suaeda glauca and *Suaeda salsa* employ different adaptive strategies to cope with saline-alkali environments. Agronomy.

[ref-38] Su WX, Xu LX, Jiang WT, Liu YL, Wang F, Lyu YR, Yan JX (2022). Effects of different exogenous substances on seed germination and seedling growth and physiology of *Perilla frutescens* under saline-alkali stress. Acta Agrestia Sinica.

[ref-39] Waheed A, Zhuo L, Wang MH, Xu HL, Tong ZW, Wang CH, Aili A (2024). Integrative mechanisms of plant salt tolerance: Biological pathways, phytohormonal regulation, and technological innovations. Plant Stress.

[ref-40] Wang WC, Zhang FH, Sun LP, Yang L, Yang Y, Wang YJ, Siddique KHM, Pang JY (2022). Alkaline salt inhibits seed germination and seedling growth of canola more than neutral salt. Frontiers in Plant Science.

[ref-41] Wu J, Chen F, Yang T, Yu H (2023). Unveiling singlet oxygen spin trapping in catalytic oxidation processes using *in situ* kinetic EPR analysis. Proceedings of the National Academy of Sciences of the United States of America.

[ref-42] Xie ZY, Chang XY, Zhao QN, Ma SR, Li YT (2023). Study on species diversity of compositae in Tongbiguan nature reserve of Yunnan Province. Eucalypt Science & Technology.

[ref-43] Xu Z (2022). Ecophysiological responses of seed germination and seedling growth to water level and saline-alkali regime in Lotus seed.

[ref-44] Xu JH, Zhao XT, Mao KT, Wang GD, Tan WM, Shu K (2021). Advances in regulatory mechanisms of seed germination under abiotic stresses. Journal of Shaanxi Normal University (Natural Science Edition).

[ref-45] Yang HW, Zhang LY, Li XH (2023). Research on the moisture content variation and influence to rice seed germination under salt and alkali stress by low field NMR. Crops.

[ref-46] Yu C, Zhang YL, Li QY, Zhou Y, Li HM, Xia Z, Huang Y (2024). Effects of saline-alkali stresses on seed germination and seedling physiological and biochemical characteristics of Origanum vulgare. Acta Agrestia Sinica.

[ref-47] Zhuang SY, Xu HB, Hu XY, Dai S, Zhang YN (2023). Effects of saline-alkali stress on growth and physiological characteristics of color leaf Heuchera micrantha ‘Silver Fan’ seedlings. Bulletin of Botanical Research.

